# Historical and Projected Impact of Global Climate Change on the Extrinsic Incubation of *Dirofilaria immitis*

**DOI:** 10.1002/ece3.72525

**Published:** 2025-12-15

**Authors:** Peter Jonathon Atkinson, Torben Dahl Nielsen, Charles Caraguel

**Affiliations:** ^1^ The University of Adelaide – Roseworthy Campus Roseworthy South Australia Australia; ^2^ The University of Sydney Camperdown New South Wales Australia

**Keywords:** canine heartworm disease, climate change, *Dirofilaria immitis*, epidemiology, extrinsic incubation, infectious disease, vector‐borne disease, veterinary science

## Abstract

Canine heartworm disease is caused by a mosquito‐transmitted filarial nematode, *Dirofilaria immitis*, and the observed heterogenous global distribution of *D. immitis* cannot be fully explained by the distribution of its vectors. Transmission of *D. immitis* requires maturation of larvae within the mosquito, requiring a sustained ambient temperature above 14°C. Lower temperatures may limit transmissibility, with areas experiencing 8 year‐round or seasonal Temperature Limited Transmissibility (TLT) reporting lower, apparently restricted, prevalence compared to areas never experiencing TLT, having the potential to become hyperendemic in the dog population and resulting in a zoonotic risk. We used weather records to investigate the effect of climate change on global *D. immitis* transmissibility since 1980 and investigated three different carbon emissions scenarios to assess the future impact of projected climate changes in the years 2040, 2070 and 2100. Since 1980, climate change has had a limited impact on the epidemiology of *D. immitis* . Areas with hyperendemic potential (never experiencing TLT) increased in land area coverage by 10.7% but had no significant increase to human population coverage, although the portion of the globe experiencing year‐round TLT has reduced significantly in land area coverage by 30.2% and human population coverage by 68.8%. Projected climate change may have an impact on *D. immitis'* epidemiology, by expanding areas with hyperendemic potential into densely populated regions, with implications for dog and human populations. These shifts are independent of mosquito range changes and may approximate redistribution of other infectious diseases with similar extrinsic incubation.

## Graphical abstract

We have used past temperature records and future temperature projections to understand the past and possible future epidemiology of canine heartworm. The range of the area hyperendemic for heartworm may change in the future, especially in extreme carbon emissions scenarios.

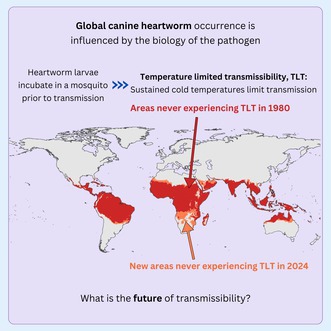

## 1 Introduction

Reports of vector‐borne diseases (VBDs) have increased faster worldwide than any other infectious diseases in recent years (Bartlow et al. 2019; Rocklöv and Dubrow 2020). Numerous anthropogenic factors have contributed to this acceleration, including climate change and increased interfaces between definitive host and vector populations, following changes in land use and urbanisation (Bartlow et al. 2019; Rocklöv and Dubrow 2020; Chala and Hamde 2021; de Souza and Weaver 2024).

Vectors are necessary for VBD transmission, and there has been much focus on the impact of climate change on altering vector ecology, and therefore potential shifts to VBD distribution (Patz et al. 2000; Campbell‐Lendrum et al. 2015; Chala and Hamde 2021; Laporta et al. 2023; de Souza and Weaver 2024). However, vector presence itself is not sufficient for transmission. Many VBD lifecycles include an extrinsic incubation period (EIP) where the infectious agent goes through necessary developmental stages within the biological vector prior to infecting a new host (de Souza and Weaver 2024).

One example of a VBD with extrinsic incubation is *Dirofilaria immitis*, the causative agent of canine heartworm disease (Kume and Itagaki 1955). Although this nematode typically infects canids (both domestic and wild), it can be an accidental zoonosis, causing pulmonary granulomatous nodules in places of hyperendemicity (McCall et al. 2008), and is closely related to the causative agents of the neglected tropical disease, lymphatic filariasis (Godel et al. 2012). Although *D. immitis* is present on all inhabited continents, its occurrence is heterogenous (Dantas‐Torres et al. 2023). It is less common, or non‐existent, in locations away from the equator where ambient temperatures are on average (or seasonally) cooler (Self et al. 2019; Atkinson et al. 2025). Whilst vectors may be more abundant closer to the equator, the heterogenous occurrence of *D. immitis* is only partially explained by the variability in *Aedes* spp. (Wang et al. 2014; Oliveira et al. 2021; Laporta et al. 2023), *Anopheles* spp. (Sinka 2013), *Ochlerotatus notoscriptus* (Peterson and Campbell 2015) and *Culex* spp. (Gorris et al. 2021; Moser et al. 2023) vectors across the Americas, Europe, and Australia.

Larvae of *D. immitis* must complete their EIP in the mosquito vector, requiring a minimum accumulation of 130 degree‐days (measured as heartworm development units; HDUs) (Slocombe et al. 1989). As mosquitoes are ectothermic, the ambient temperature dictates the length and completion of the EIP ‐ that is, it takes longer in cooler weather, and is interrupted once the ambient temperature drops below 14°C (Christensen and Hollande 1978; Fortin and Slocombe 1981). If the EIP is not completed within the mosquito vector's lifespan, a maximum of 30 days for *D. immitis* vectors (Slocombe et al. 1989), the life cycle is broken and transmission is not possible. Therefore, beyond the vector activity alone, the occurrence of *D. immitis* is bounded by its transmissibility, determined by its EIP completion. This involves necessary HDU accumulation and, ultimately, sustained warm ambient temperature (Brown et al. 2012; Cuervo et al. 2015; Lavallée‐Bourget et al. 2024). Areas with no seasonal limitations to EIP completion reported unrestricted infection occurrence (i.e., up to 100%), and areas experiencing seasonal EIP limits reported lower and apparently restricted occurrence (Atkinson et al. 2025).

Recently, there have been increasingly frequent reports of canine heartworm in parts of Europe where it was rarely diagnosed previously (Genchi et al. 2011; Fuehrer et al. 2021; Morchón et al. 2022), as well as increasing prevalence in parts of North America (Self et al. 2019). Although, several factors have been cited, including the emergence of preventative‐resistant strains of *D. immitis* in the USA (Geary et al. 2011) or the increasing movements of people and their pets globally (Jahn et al. 2023), climate change is also speculated to be a driver (Genchi et al. 2011; Fuehrer et al. 2021), with a so far unmeasured impact on the global *D. immitis* epidemiology. In this report, we investigated the global impact of changing ambient temperatures on the likelihood of *D. immitis* to complete its EIP. Using historical temperature records starting in 1980, we found where EIP completion was not limited by cooler ambient temperatures worldwide, and therefore at risk of unrestricted occurrence, describing its progression through the relative land area and human population (as a proxy of dog population) coverage. We also predicted the future coverage of these areas using temperature projections from carbon emission scenarios ranging from a sustainable pathway to unrestricted fossil fuel development.

## 2 Materials and Methods

### 2.1 Historical Distribution of Temperature Restricted *Dirofilaria immitis* Extrinsic Incubation Period

We define here Temperature Limited Transmissibility (TLT) as a scenario when ambient temperatures at a given location and time did not meet the minimum EIP requirements of *D. immitis* . In TLT areas and times, transmission of *D. immitis* would not be possible, regardless of the distribution of the mosquito vector or proximity to an infected individual. A decrease to either the spatial and temporal coverage of TLT, would indicate a potentially larger area where, or longer time when, *D. immitis* transmission may occur. We mapped TLT areas of the globe using historical maps of daily minimum and maximum temperature records from 1980 to 2024, following a similar method as described by Atkinson et al. (2024a).

First, we created daily heartworm development unit (HDU) maps representing, at each location for a given day, the cumulated count of degree days by which the ambient temperature exceeded 14°C. We retrieved minimum and maximum daily temperature records for all days from 2nd December 1979 to 31st December 2024 (inclusive) from the National Oceanic and Atmospheric Administration's Physical Sciences Laboratory database (NOAA PSL 2024) provided as a raster file, at a resolution of 0.5 × 0.5°(each containing 259,200 cells). These records represent the near‐surface air temperature (approximately two metres above ground level) of cells corresponding to a land mass (i.e., provide a missing value at locations over major water bodies) with the exclusion of Antarctica. We applied the Baskerville–Emin method to model the daily sinusoidal fluctuation between a day's minimum and maximum temperature and calculate the daily accumulable HDUs available at each raster cell (Baskerville and Emin 1969). Across the 16,467 days within the study period, weather records were missing for 24 days (the list of missing days is accessible at https://psl.noaa.gov/). For these days, we took the mean of the minimum and maximum temperature, respectively, of the days immediately preceding and following the missing day, to interpolate the missing day's minimum and maximum temperature, and produced the daily HDU map using these values.

We then generated daily rasters to represent the total cumulated HDU (cHDU) for each raster cell over the preceding 30 days. Thirty days represents the most likely maximum lifespan of mosquito vectors incubating *D. immitis* (Slocombe et al. 1989), and is routinely used to model *D. immitis* ' EIP (Perles et al. 2024). For this, we summed each cell's daily HDU rasters over the prior 30 days of each day between 1st January 1980 and 31st December 2024 (inclusive).

Each daily cHDU map was then binarised to create a daily TLT map, by classifying regions where EIP could not be completed and, therefore, where transmission was not possible, based on degree day accumulation of < 130 HDUs. Using the daily TLT maps, we produced yearly TLT maps to show the count of days each raster cell experienced TLT, ranging between 0 and 365 days (366 days in leap years). Each yearly TLT map was categorised into TLT zones, reporting EIP disruption either (i) year‐round (all days experienced TLT), (ii) seasonally (at least 1 day of the year experienced TLT) or (iii) never (i.e., every day of a given year was warm enough to complete EIP, so TLT did not occur).

Using these yearly TLT zonal maps, we calculated the proportion of (i) land area and (ii) 2020 human population living in each TLT zone. The human population raster was accessed through the Socioeconomic Data and Applications Center (Center for International Earth Science Information Network ‐ CIESIN ‐ Columbia University 2020), provided at a resolution of 0.00833 × 0.00833° (containing 933,120,000 cells). To find the proportion of the population living within each TLT zone, we first aggregated (by summation) the population raster to a resolution of 0.5 × 0.5°to match the yearly TLT zonal map resolution. Then, we overlaid it on each yearly TLT zonal map to find the proportional coverage of the population living in each TLT zone.

#### 2.1.1 Historical Changes to TLT Zoning

We aimed to assess sustained changes to TLT zoning across the study period and performed pairwise and stepwise comparisons between consecutive years throughout the entire study. Briefly, we started by comparing the TLT zone distribution from 1981 with 1980 and highlighted any cells that changed TLT zones between these years. Then, we repeated this comparison for each successive pair of years until 2024. We summed pairwise changes across the entire study period, to ensure only cells with a net change to their TLT zone remained. Those cells with no overall change indicated either (i) an unchanged TLT zone for the entire period or (ii) no net change to the TLT zone (for instance, if a cell flipped equally between TLT zones, the net change would be zero).

#### 2.1.2 Historical Changes to Yearly Count of TLT Days

To better understand TLT zone changes, we also summarised the net change of the yearly count of TLT days over the study period. Starting with 1980 and 1981, we calculated the pairwise difference in count of TLT days at each raster cell (ranging from 0 to 365, or 366 in leap years). We repeated this pairwise calculation until 2024 and summed the net difference to generate a raster visualising the worldwide trend. A net change of zero indicated either (i) an unchanged count of TLT days or (ii) pairwise changes averaging zero over the study period. A net negative count for a cell indicated a decrease in TLT days per year at a given cell (overall warming effect), and a positive count indicated an increase in TLT days (overall cooling effect).

#### 2.1.3 Time‐Series Analysis of TLT Zone Changes

We performed simple linear regressions to estimate the effect of time (modelled in years as a continuous predictor) on the yearly proportional coverage (of land area or population) of each of the three TLT zones (deemed continuous outcomes); that is, either ‘year‐round’, ‘seasonal’ or ‘never’. Assumptions of linear regression (linearity, homoscedasticity and normality of residuals) were assessed as described by Dohoo et al. (2014).

### 2.2 Predictions of TLT Zonal Distribution

We predicted and compared TLT zonal distribution from present‐day to 2100 using three shared socio‐economic pathway (SSP) projections, representing three different carbon emissions scenarios. We sourced daily minimum and maximum temperature projections from the World Climate Research Programme repository, featured in the Intergovernmental Panel on Climate Change's sixth assessment report (Eyring et al. 2016), downloadable at https://esgf.nci.org.au/search/cmip6‐nci/ (WCRP CMIP6, n.d.). We elected to use a projection from the CSIRO's ACCESS‐CM2 programme, model variant r1i1p1f1, and accessed data produced from the scenarios presented in SSP1‐2.6, SSP2‐4.5 and SSP5‐8.5, to represent ‘sustainability’, ‘middle of the road’ and ‘fossil fuel development’ carbon emission scenarios, respectively. These provided predicted daily temperature maximum and minimum rasters from 2015 to 2100 at a resolution of 1.875 × 1.25° (containing 27,648 cells) for all cells of the globe, including those at locations over oceans and the continent of Antarctica. Therefore, we cropped them to only retain values corresponding to those available in the yearly TLT zonal map for 2024. Yearly TLT zone maps for each SSP scenario were made for 2016–2024, and three future calendar years of 2040, 2070 and 2100, by following the steps described in Section 2.1.

#### 2.2.1 Predicted Changes to TLT Zoning

Since historical temperature records may contain variability that cannot be perfectly modelled in predicted temperatures, we expected discrepancies between the TLT zonal coverage estimates of the historical and predicted maps (when both were available i.e., 2016–2024). Therefore, comparing future predicted TLT zones to historical TLT zones may have included systematic departures due to unpredictable variability. Therefore, we elected to compare our future predicted TLT zones to predicted TLT zones from 2016 to 2024.

To do this, we first generated a reference TLT zonal map for each SSP scenario, reflecting the TLT zoning as predicted by the SSP data for the years 2016–2024. Using predicted data from each SSP scenario, we calculated predicted TLT maps for these years (nine maps, covering 2016–2024). We then summarised the predicted TLT maps into a reference TLT map, taking the median count of TLT days for each cell. As previously, we zoned each reference map with three areas experiencing TLT either ‘year‐round’, ‘seasonally’ or ‘never’. This reference map was later used to benchmark predicted future TLT zone distributions generated for 2040, 2070 and 2100 in each corresponding SSP scenario.

For each SSP scenario and using the corresponding TLT reference map, we mapped the net difference in both TLT zone and yearly count of TLT days between the predicted years of interest (2040, 2070 or 2100) and the corresponding reference map, as described in Sections 2.1.1 and 2.1.2.

#### 2.2.2 Trends to Changes to Transmissibility Zoning

For each predicted TLT zonal map (reference, and 2040, 2070 and 2100), we calculated the proportional coverage of each TLT zone (year‐round, seasonal and never) in terms of land area and 2020 human population. We used our reference TLT zonal coverage map (generated in Section 2.2.1) as a benchmark to identify and account for unmodelled temperature variability. We first estimated the relative systematic departure between historical and projected maps, for each predicted TLT zone. We found the median ratio of zonal coverage in the reference TLT map to the historical TLT map of the corresponding year, for the 2016 to 2024, that is, $\frac{\text{projected}\;\left[\mathrm{TLT}\;\text{zone}\right]\;\text{coverage}}{\text{historical}\;\left[\mathrm{TLT}\;\text{zone}\right]\;\text{coverage}}$. Next, we applied this correction factor to the predicted coverage estimates for the years 2040, 2070 and 2100, by dividing the estimated proportional coverage value by its correction factor, that is, $\frac{\text{projected}\;\left[\mathrm{TLT}\;\text{zone}\right]\text{coverage}}{\text{correction factor}\;\left[\mathrm{TLT}\;\text{zone}\right]}$, repeating this for each SSP scenario. This provided us, with predicted TLT zonal coverage estimates that were directly comparable with the historical coverage values.

All mapping and analyses were performed in R version 4.4.1 (R Core Team 2025) using functionality from the contributed *raster*, *terra*, *sf*, *sp*, *maps*, *ncdf4*, *tidyr* and *gvlma* packages (Pebesma and Bivand 2005; Pebesma 2018; Pena and Slate 2019; Becker et al. 2022; Hijmans 2022a, 2022b; Pierce 2023). Our R script is available at http://doi.org/10.5061/dryad.qv9s4mwsb.

## 3 Results

We are reporting 54 yearly Temperature Limited Transmissibility (TLT) zonal maps (1980–2024, and three each for 2040, 2070, 2100), which categorised areas worldwide into the three TLT zones ‐ that is, ambient temperatures not meeting *Dirofilaria immitis*' EIP requirements either (i) year‐round (every day of a given year), (ii) seasonally (at least 1 day of the year) or (iii) never (none of the days). These maps are available at https://doi.org/10.5061/dryad.qv9s4mwsb.

### 3.1 Current Global Distribution of TLT Zones

**FIGURE 1:**
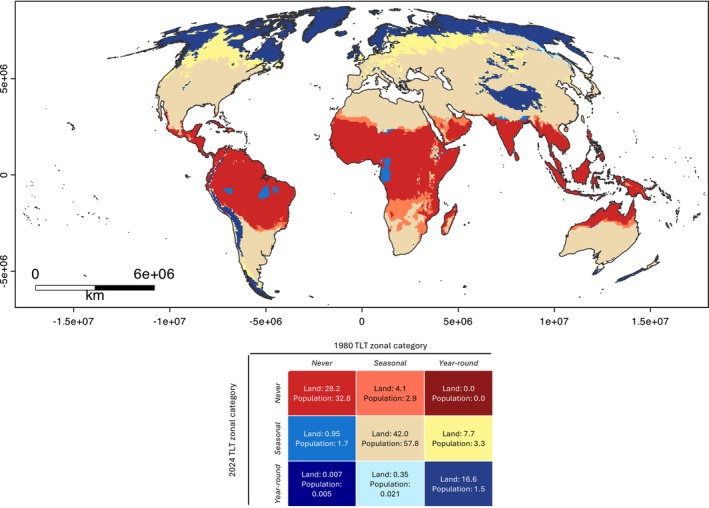
Temperature Limited Transmissibility (TLT) zoning of *Dirofilaria immitis* (year‐round, seasonal or never) for 1980 and net reclassification of TLT zone category to 2024, based on temperature‐dependent completion of the extrinsic incubation period. Map presented in the Mollweide (EPSG:54009) projection with coastal borders shown. Percentage of land area (L) and 2020 human population (P) affected by reclassification are reported within each colour‐coded zone.

The current distribution of the year‐round TLT zone was generally at the extreme bounds of latitude (beyond 50°or −50°) and covers approximately a fifth of the land area and < 5% of the population (Figure 1, Table 1). The never TLT zone occurred at latitudes approximating the tropical belt and covered roughly a third of the land area and population. The seasonal TLT zone was the largest, covering approximately half of the land area and two‐thirds of the population.

**TABLE 1:** Median percentage coverage of global land area and 2020 human population where the ambient temperature did not meet the requirements for *Dirofilaria immitis* extrinsic incubation period either year‐round (every day of a given year), seasonally (at least 1 day of the year) or never (none of the days) from 1980 to 2024.

	Median of yearly global land area % coverage (range)	Median of yearly global human population % coverage (range)
Year‐round	20.5 (16.9–24.8)	2.38 (1.34–4.84)
Seasonal	49.9 (46.2–52.8)	63.6 (60.6–67.4)
Never	29.5 (27.1–32.4)	33.9 (30.5–36.8)

### 3.2 Historical Variations in the Global Distribution of TLT Zones

#### 3.2.1 Changes to TLT Zoning

Most of the locations (86.8%) and population (92.1%) had no sustained change in their TLT zone between 1980 and 2024 (Figure 1). For the rest of the locations (13.2%), the vast majority (11.8%) moved to a lower TLT category—either from year‐round to seasonally (7.7%, mostly in northern North America and Europe) or from seasonally to never (4.1%, mostly in southern Africa and the Arabian Peninsula), which covered approximately 6.2% of the total 2020 human population. A small number of locations (1.3% of land area) saw their TLT zoning increase, mostly from never to seasonally (0.95%, mainly in the Amazon rainforest and western Africa), covering only 1.7% of the population. The relative size of the year‐round TLT zone shrunk by 30.2%, although this area covered 68.2% of the population originally living in this zone. The seasonal and never TLT zones grew by 9.0% and 10.7%, respectively, increasing the population living in these zones by 3.4% each.

#### 3.2.2 Changes to Yearly Count of TLT Days

**FIGURE 2:**
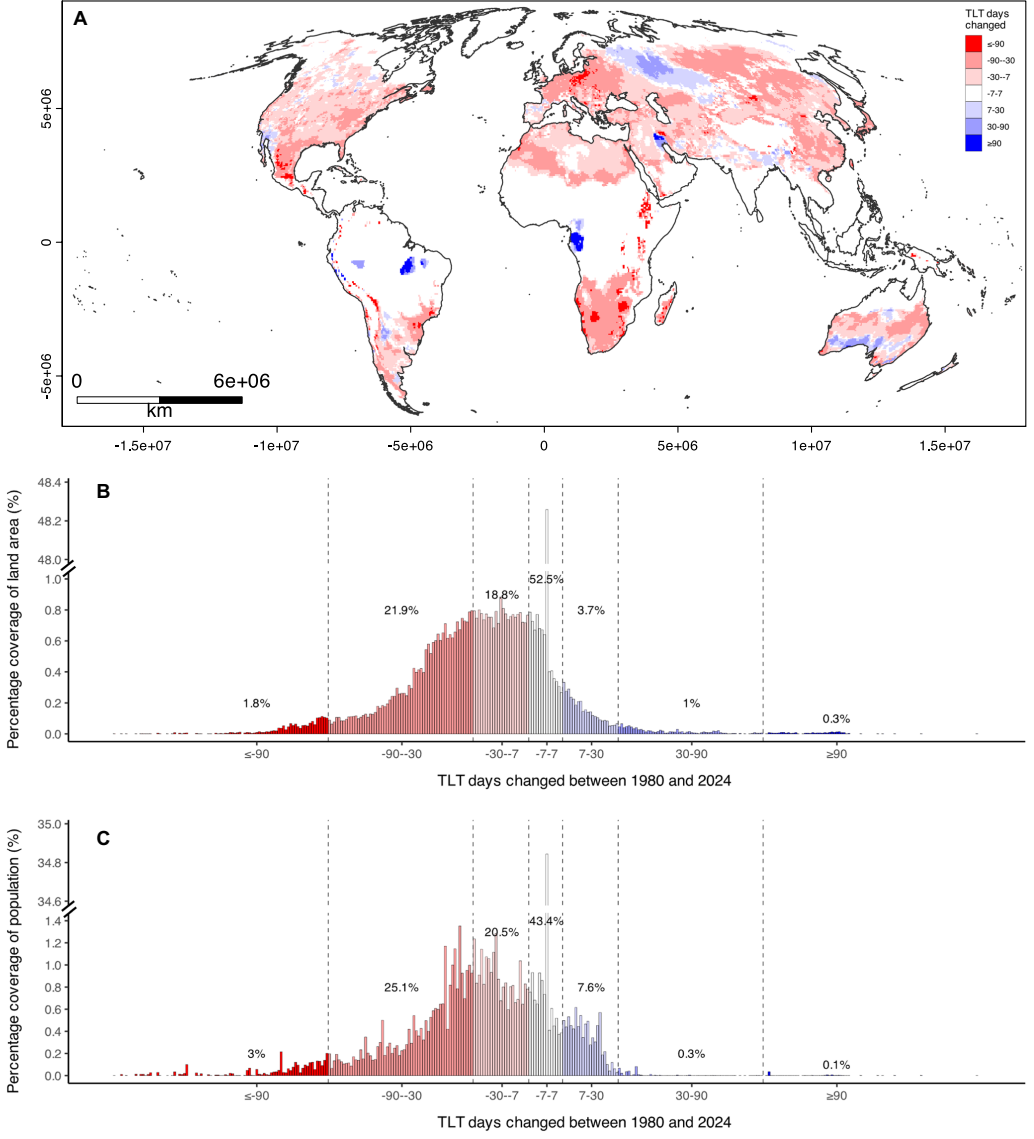
Net change in Temperature Limited Transmissibility (TLT) days per calendar year supporting *Dirofilaria immitis* transmission from 1980 to 2024. (A) Global map of changes, (B) density histogram by land area, and (C) density histogram by 2020 human population. Warm colours indicate a net decrease in TLT days (reduced restriction of transmission), while cool colours indicate a net increase (greater restriction of transmission). White areas represent little or no change. Maps are shown in Mollweide projection (EPSG:54009) with coastal borders outlined. Note the broken vertical axis, and that the horizontal axis of each histogram (B and C) is bounded at ±140 days to aid visualisation.

Figure 2 shows the net change to the yearly count of TLT days from 1980 to 2024. The median net change between 1980 and 2024 was zero days but ranged from −345 to 366 days. Approximately half of the global land area saw no or little net change in the yearly count of TLT days between 1980 and 2024 (±7 days across 44 years), while two fifths experienced a noticeable decrease (> 7 days) and only 5.1% experienced an increase (< 7 days) (Figure 2). Changes to population coverage of TLT days followed a similar trend, although more of the population experienced both an increase and decrease in TLT days than for land area, resulting in less than 50% of the population experiencing no or little net change (±7 days) in TLT days.

#### 3.2.3 Trends to Changes to TLT Zoning

**FIGURE 3:**
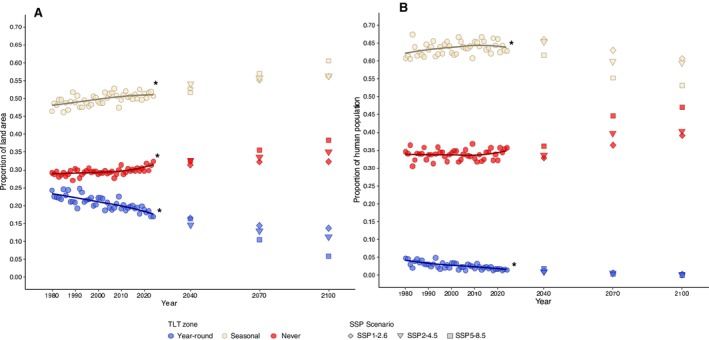
Scatterplots and locally estimated scatterplot smoothing regression lines of the proportions of land area (A) and 2020 human population (B) included in the Temperature Limited Transmissibility (TLT) zones of *Dirofilaria immitis* each year, based on temperature‐dependent completion of the parasite's extrinsic incubation period. Lines marked with a * indicate significant changes to the coverage of the zone with time. TLT zones are split into those restricting transmission year‐round (blue), seasonally (cream) or never (red). Projected temperature data from three selected shared‐socio‐economic pathways (SSPs) of climate change were used to generate projected transmissibility zones for the years 2040, 2070 and 2100—*SSP1‐2.6*: ‘sustainability’, *SSP2‐4.5*: ‘middle of the road’ and *SSP5‐8.5*: ‘fossil fuel development’ carbon emissions.

Apart from the analysis of human population coverage in the ‘year‐round’ TLT zone, all regressions satisfied the modelling assumptions. This model had significantly heteroscedastic residuals (*p* = 0.006) most likely due to zero bounding of lower percentages. We found a significant negative effect of time on the proportion of land area where the TLT was year‐round, that is, this zone significantly shrunk by 1.20% (95% CI: 0.92%–1.50%) each decade (Table 2, Figure 3A). During the same period, the seasonal TLT zone was estimated to grow by 0.76% per decade (95% CI: 0.46%–1.10%) and the never TLT zone grew slightly less by 0.45% per decade (95% CI: 0.25%–0.65%). We observed similar trends in the changes to the proportional coverage of the human population (Table 2, Figure 3B), however, the population coverage did not increase significantly in the never TLT zone (*p* = 0.56), that is, this zone extended in poorly populated areas (as of 2020).

**TABLE 2:** Estimated regression coefficients of the effect of a 10‐year increase on the percentage coverage of global land area or 2020 human population living in a zone where the ambient temperature did not meet the requirements for *Dirofilaria immitis* extrinsic incubation period either year‐round (every day of a given year), seasonally (at least 1 day of the year) or never (none of the days) between 1980 and 2024.

	Change in % global land area (95% CI)	Change in % global human population (95% CI)
Year‐round	−1.20 (−1.5, −0.92)	−0.53 (−0.68, −0.37)
Seasonal	0.76 (0.46, 1.1)	0.43 (0.030, 0.82)
Never	0.45 (0.25, 0.65)	0.10 (−0.24, 0.44)

*Note:* 95% confidence intervals for each coefficient are shown in parentheses.

### 3.3 Projected Variation in *D. immitis* Temperature Bounded Transmissibility Distribution

#### 3.3.1 Predicted Changes to TLT Zoning

**FIGURE 4:**
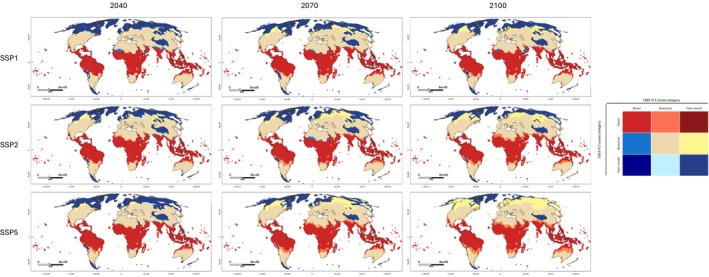
Predicted impact of climate change on the Temperature Limited Transmissibility (TLT) of *Dirofilaria immitis* zoning (year‐round, seasonal or never). TLT in 2040, 2070 and 2100 is compared to median TLT zonal classification from 2016 to 2024. Colour coding of zonal changes is provided, and maps are presented in the Mollweide (EPSG:54009) projection with coastal borders shown.

Figure 4 shows the expected change to TLT zones (year‐round, seasonal or never) from present day to future years. Changes to TLT zones were on the borders between zones, with a trend to increase in a downward direction ‐ that is, year‐round to seasonal (mostly in the northern hemisphere) or seasonal to never (mostly in the southern hemisphere). The most extreme carbon emissions scenario (SSP5‐8.5) was predicted to result in the most substantial changes to TLT zoning, particularly by 2100, with almost complete replacement of the year‐round TLT zone with the seasonal zone (Figure 4).

#### 3.3.2 Predicted Changes to Yearly Count of TLT Days

**FIGURE 5:**
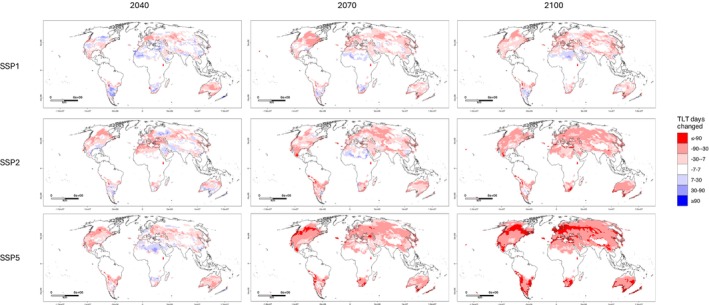
Net change of Temperature Limited Transmissibility (TLT) days of *Dirofilaria immitis* per calendar year from present day to years 2040, 2070 and 2100, based on projected daily weather from three shared socio‐economic pathways (SSPs) of climate change. Areas shaded warm colours indicate a net decrease to the count of TLT days, and areas shaded in cold colours indicate a net increase to the count of TLT days. Areas in white indicate no or little change to TLT. Maps are presented in the Mollweide (EPSG:54009) projection with coastal borders shown. *SSP1‐2.6*: ‘sustainability’, *SSP2‐4.5*: ‘middle of the road’ and *SSP5‐8.5*: ‘fossil fuel development’.

Figure 5 maps the predicted changes to the yearly count of TLT days between present day and 2040, 2070 and 2100. Most changes to predicted TLT days were to decrease across the northern hemisphere and Australia (for SSP1‐2.6), as well as South America and southern Africa for SSP2‐4.5 and SSP5‐8.5 (Figure 5). However, there were pockets of increasing TLT days across parts of northern Africa, the Americas, Europe and Asia in the SSP1‐2.6 scenario, and in 2040 in the SSP2‐4.5 scenario. Substantially more of the globe was projected to experience a decrease of at least 90 TLT days in the SSP5‐8.5 scenario than in other scenarios (Figure 5).

#### 3.3.3 Trends to Changes to TLT Zoning

Projected changes to the land coverage of each TLT zone followed historical trends. The proportion of land area in the never and seasonal TLT zones continued to increase, with a corresponding decrease in the zone always experiencing TLT, regardless of the projection scenario (Figure 3A). We observed marginal differences between the three selected carbon emissions models, except for the unrestricted carbon emission scenario (SSP5‐8.5), showing a faster progression toward 2100.

The proportion of the 2020 human population living in the year‐round TLT zone was projected to continue to decrease, although it was already at a residual level (Figure 3B). The proportional coverage of the population living in the zone never experiencing TLT was projected to increase more rapidly than observed between 1980 and 2024, resulting in a corresponding decrease in the coverage of the population in the seasonal zone. The most extreme changes were again observed with the SSP5‐8.5 scenario projections.

## 4 Discussion

We used the temperature‐bounded development of *D. immitis* in its mosquito vector to investigate and describe its global transmissibility from 1980 to 2024 and used predicted temperature data from three global carbon emissions scenarios to investigate the impacts of future climate change. We reported Temperature Limited Transmissibility (TLT) as areas where, or days when, recent ambient temperatures would not support the completion of *D. immitis'* EIP and therefore where transmission would not be possible (regardless of other necessary factors, such as vector or reservoir presence) (Knight and Lok 1995; Brown et al. 2012). For all included years, TLT was heterogeneous across the globe. Some areas experienced TLT year‐round (all days of the year), some seasonally (at least 1 day of the year) and some never (i.e., every day of a given year was warm enough to complete EIP, so TLT was never experienced).

Higher prevalence of *D. immitis* infection in dogs has been reported in areas experiencing fewer TLT days, such as in southern USA (Wang et al. 2014), northern Australia (Atkinson et al. 2025), northern South America (Labarthe et al. 2014), India (Borthakur et al. 2015) and southern Europe (Montoya et al. 1998; Tahir et al. 2017). Recently, a serological survey of cats reported higher prevalence in areas with less TLT (Genchi et al. 2024). Human *D. immitis* infections tend to occur in similar areas, such as southeastern USA, Brazil and the Mediterranean region of Europe (Simón et al. 2012). Reported *D. immitis* prevalence in dogs tends to be lower in locations such as northern USA and Canada (Wang et al. 2014; Evason et al. 2019; McGill et al. 2019), southern Australia (Atkinson et al. 2025), southern South America (Vezzani and Carbajo 2006; Vezzani et al. 2011) and other parts of Europe (Morchón et al. 2022) where we found TLT was experienced for a larger proportion of each year, and the reports of human infections in these areas are either sporadic or absent (Simón et al. 2012). As suitable climate is necessary, but not sufficient for disease transmission (Rocklöv and Dubrow 2020), our work demonstrates that TLT (disrupted transmission) may result in an upper limit to prevalence in the dog population (i.e., it becomes saturated), and potential hyperendemicity (unrestricted prevalence and possible zoonoses) results if weather conditions never limit transmission.

Understanding changes to the zone never experiencing TLT is therefore necessary to appreciate possible future changes to the global epidemiology of *D. immitis* . Until now, expansion of this zone occurred in relatively sparsely populated areas, therefore having a limited effect on *D. immitis'* epidemiology in both dog and human populations. However, predictions suggest that its continued expansion will occur in more densely populated areas between 2040 and 2070, continuing to 2100 (Figure 3). These changes result in an expansion of areas potentially hyperendemic for *D. immitis* in parts of South America, northern sub‐Saharan and southern Africa, southern and southeast Asia and northern Australia (Figure 4). Veterinarians working in these regions should enhance monitoring efforts to document changes to *D. immitis* epidemiology in the dog population, and local health authorities should be made aware of the increased zoonotic risk.

Our work also highlights the substantial land area and human population living in areas experiencing seasonal TLT (Figure 1). Some of these areas, such as the USA, Australia and Europe have current recommendations for year‐round preventative use in dogs (Korman et al. 2014; ESDA 2017; AHS 2024). Based on our TLT zoning, year‐round preventative administration may not be necessary to prevent canine heartworm infections, and therefore, dirofilariosis for many pet owners. Instead, veterinarians may elect to educate pet owners about the impact of weather restrictions on transmissibility, allowing owners to opt for a risk‐based preventative strategy. Despite the acknowledgement of possible seasonal transmission, the justification for year‐round prevention is often to enhance owner compliance (Korman et al. 2014; Ketzis and Epe 2023; AHS 2024) or due to the impact of heat islands disabling TLT for longer, unmeasurable periods (Korman et al. 2014; ESDA 2017). However, we believe the risk of *D. immitis* infection may be over‐inflated in practice guidelines, resulting in veterinarians recommending owners take a risk‐averse, year‐round preventative program where this may not be necessary. This problem may be further pronounced as some practice guidelines are funded by parties with financial conflicts of interest, and some veterinarians may be financially motivated to make these recommendations. Ultimately, a preventative strategy needs to be agreed upon by both the pet owner and their veterinarian after sound advice, taking into consideration not only an individual dog's inherent risk of infection, but also the dog owner's values and preferences. A risk‐based preventative plan may not be feasible in some veterinary settings, and risk assessment is not always possible. However in Australia, real time monitoring of the TLT of *D. immitis* is currently being performed (Atkinson et al. 2024b), equipping veterinarians with the ability to educate owners about when and where weather disrupts transmission, facilitating a risk‐based preventative plan if appropriate.

Notably, there was a mismatch between the projected and historical transmissibility maps for 2016–2024, with the projections tending to underestimate areas experiencing TLT never or seasonally (data not shown). This highlights the rate of climate change, and its warming effects may be increasing faster than IPCC projections suggest (Schmidt 2024; Hansen et al. 2025). This also emphasises the need for ongoing monitoring of TLT, as the currently static outputs we present here may change in the future.

Our work has some potential limitations. Firstly, since we used the 2020 human population to proxy the domestic dog population, our inferences to the impacts of *D. immitis* occurrence in dogs rely on the assumption that the dog population is proportional to the human population. However, the human population has been used as a proxy for the dog population previously (Aegerter et al. 2017; Atkinson et al. 2024a), and reflects the dog population that may have access to veterinary care and advice. We also analysed our results taking the population distribution as a static measure, despite its probable shift with climate change (Xu et al. 2020). Finally, our work was sensitive to the collection, collation and reporting of the global weather data. It is likely that some collection sites have been added or removed since 1980 which would affect the accuracy of the interpolation. For instance, in the Amazon rainforest and western Africa, there was seemingly an increase in TLT zoning from never to seasonal since 1980 (Figure 1) and we suspect this to be a data artefact rather than a true change. However, due to the large number of data points collected over a long study period, we expect the impact of changes to coverage to not have affected the interpretation of our study's results.

## 5 Conclusion

We used *D. immitis* as a case example to illustrate the effects of climate change on the distribution and prevalence of a VBD. Our study highlights a currently limited epidemiological impact of climate change on the temperature‐limited transmissibility of *D. immitis* . However, we predict future changes to have a more pronounced impact, decreasing the disruption to transmission as carbon emissions increase, therefore increasing its possible transmission. This may have impacts in both the dog and human populations by increasing the coverage of areas with hyperendemic potential. These changes may also reflect a simultaneous increase in the transmissibility of other VBDs with extrinsic incubation, such as malaria, dengue fever, lymphatic filariasis and yellow fever (Bancroft 1906; Bauer and Hudson 1928; Nikolaev 1935; Christensen and Sutherland 1984; Johansson et al. 2010; Kamiya et al. 2020; Stopard et al. 2021), and efforts should be made to document and monitor the TLT of these pathogens.

## Author Contributions

**Peter Jonathon Atkinson:** conceptualization (lead), data curation (lead), formal analysis (lead), investigation (lead), methodology (lead), project administration (lead), software (lead), visualization (lead), writing – original draft (lead), writing – review and editing (lead). **Torben Dahl Nielsen:** conceptualization (supporting), data curation (supporting), formal analysis (supporting), investigation (supporting), methodology (supporting), project administration (supporting), software (supporting), supervision (equal), visualization (supporting), writing – original draft (supporting), writing – review and editing (supporting). **Charles Caraguel:** conceptualization (supporting), data curation (supporting), formal analysis (supporting), investigation (supporting), methodology (supporting), project administration (supporting), software (supporting), supervision (equal), writing – original draft (supporting), writing – review and editing (supporting).

## Conflicts of Interest

The authors declare no conflicts of interest.

## Data Availability

Data associated with this manuscript is either in the public domain (with references provided) or accessible at https://doi.org/10.5061/dryad.qv9s4mwsb.
